# Adipose tissue-derived stem cells as a therapeutic strategy for enterocutaneous fistula: an experimental model study

**DOI:** 10.1590/acb384523

**Published:** 2023-10-13

**Authors:** Vitor Penteado Figueiredo Pagotto, Cristina Pires Camargo, Paula Vitória Cáceres, Silvana Cereijido Altran, Rolf Gemperli

**Affiliations:** 1 Universidade de São Paulo – Faculdade de Medicina – Disciplina de Cirurgia Plástica – São Paulo (SP) – Brazil.; 2 Universidade de São Paulo – Faculdade de Medicina – Hospital das Clínicas – Serviço de Cirurgia Plástica – São Paulo (SP) – Brazil.; 3 Universidade de São Paulo – Faculdade de Medicina – Laboratório de Investigação Médica – São Paulo (SP) – Brazil.

**Keywords:** Mesenchymal Stem Cells, Wound Healing, Cutaneous Fistula, Intestinal Fistula, Surgery, Plastic, Tissue Engineering

## Abstract

**Purpose:**

Enterocutaneous fistula (ECF) is a condition in which there is an abnormal connection between the intestinal tract and the skin. It can lead to high morbidity and mortality rates despite the availability of therapeutic options. Stem cells have emerged as a potential strategy to treat ECF. This study aimed to evaluate the effect of adipose tissue-derived stem cells (ASC) on ECF in an experimental model.

**Methods:**

ECF was induced in 21 Wistar rats, and after one month, they were divided into three groups: control group (C), culture medium without ASC group (CM), and allogeneic ASC group (ASC). After 30 days, the animals underwent macroscopic analysis of ECF diameter and histopathological analysis of inflammatory cells, tissue fibrosis, and vascular density.

**Results:**

The study found a 55% decrease in the ECF diameter in the ASC group (4.5 ± 1.4 mm) compared to the control group (10.0 ± 2.1 mm, p = 0.001) and a 59.1% decrease in the CM group (11.0 ± 4.3 mm, p = 0.003). The fibrosis score in the ASC group was 20.9% lower than the control group (p = 0.03). There were no significant differences in inflammation scores among the three groups.

**Conclusions:**

This study suggests that ASC treatment can reduce ECF diameter, and reduction in tissue fibrosis may be a related mechanism. Further studies are needed to understand the underlying mechanisms fully.

## Introduction

Enterocutaneous fistula (ECF) is a medical condition characterized by an abnormal connection between the intestinal tract and the skin. It can lead to serious complications such as abdominal infections, necrosis, malnutrition, and mortality rates of up to 30%^1^. Surgical complications are responsible for 75% of ECF cases, including dehiscence of intestinal anastomoses, trophic lesions of the intestinal wall, and surgical trauma during lysis of adhesions in patients with neoplasms and inflammatory diseases^2^.

ECF management requires a multidisciplinary approach based on patient’s characteristics and the severity of the condition^1^,^3^-^8^. The ECF care protocol involves managing sepsis and skin care, providing nutritional support, defining the intestinal anatomy, and developing a surgical procedure to deal with the fistula^9^.

Initially, systemic drugs are used to reduce intestinal flow, which may include H2 receptor antagonists, proton pump inhibitors, antimotility agents, somatostatin and its analogues, depending on ECF location^10^-^15^. When the etiology of the fistula is an inflammatory bowel disease (e.g., Crohn’s disease), immunosuppressant drugs such as azathioprine and cyclosporine may be used as a pharmacologic strategy^16^. Surgery to resolve ECF should be considered only when the patient has adequate nutritional status, including albumin level above 3 g/dL and body mass index above 20 kg/m^2^, and after the resolution of any infectious process, typically after four to six weeks of observation^2^,^17^-^19^.

ECF is a condition for which no therapy providing satisfactory and permanent results is available. However, treatment with adipose tissue-derived stem cells (ASC) is emerging as a promising option for patients who do not respond to classical treatments or have restrictions for receiving immunobiological agents^20^,^21^. ASC have the ability to modulate inflammatory and immune response, making them an ideal tool in regenerative medicine^22^-^24^. Studies have shown that the mechanisms of action of ASC involve both cell proliferation and differentiation, as well as a paracrine effect through the release of cytokines, interleukins, and growth factors^25^,^26^.

This study aimed to assess the impact of the perifistular application of ASC in the treatment of ECF.

## Methods

We conducted a study on 21 male Wistar rats aged 8 to 12 weeks. The rats weighed between 200 and 250 g and were kept in species-specific cages at a temperature of 24°C, with 12/12-hour day-night cycles and *ad libitum* diet. Our project followed the recommendations for laboratory animal handling established by the National Council for the Control of Animal Experimentation and the ARRIVE guidelines^27^. The Animal Research Ethics Committee of our institution approved the study on May 14, 2019, with registration number 1,278/2019.

### Preparation of adipose tissue-derived stem cells

Three Wistar rats were anesthetized with ketamine hydrochloride (Pfizer, United States of America) at the dose of 100 mg/kg and xylazine hydrochloride (Syntec, Brazil) at the dose of 10 mg/kg. Adipose tissue was harvested from the abdominal region and enzymatically digested at 37°C using collagenase IA 0.1% dissolved in Dulbecco’s modified Eagle’s medium (DMEM, Sigma Aldrich, Germany). The material was filtered and centrifuged at 2,000 rpm for 5 minutes, and the stromal vascular fraction was isolated in culture medium comprised of 80% DMEM, 20% fetal bovine serum (Sigma Aldrich, Germany), and antibiotics (penicillin from Sigma Aldrich, Germany, streptomycin from Biofarm, Brazil, and amphotericin B from Cristalia, Brazil). The cells were then incubated at 37°C. Upon reaching 80% confluence, the cells were released using trypsin (Sigma Aldrich, Germany) and EDTA (Sigma Aldrich, Germany).

**Figure 1:**
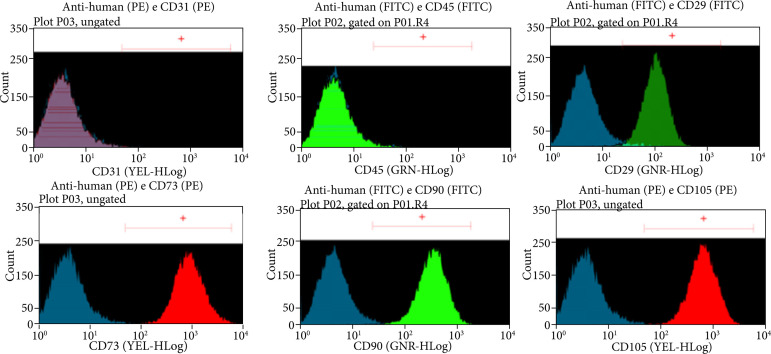
Result of flow cytometry. Markers CD31 and CD45 showed a negative result, while markers CD29, CD73, CD90, and CD105 showed a positive result.

Source: Elaborated by the authors.

The presence of ASC was confirmed, and their immunophenotyping was determined through flow cytometry assays. The CD31 and CD45 markers showed a negative result, while the CD29, CD73, CD90, and CD105 markers showed a positive result, confirming the isolation of viable ASCs. These results are shown in Fig. 1. The confirmation of viable ASCs is in accordance with the criteria established by the International Society for Cellular Therapy^28^.

### Induction of enterocutaneous fistula

Animals were submitted to the surgical procedure anesthetized with ketamine hydrochloride (Pfizer, United States of America) at the dose of 100 mg/kg and xylazine hydrochloride (Syntec, Brazil) at the dose of 10 mg/kg to create an ECF^29^.

**Figure 2:**
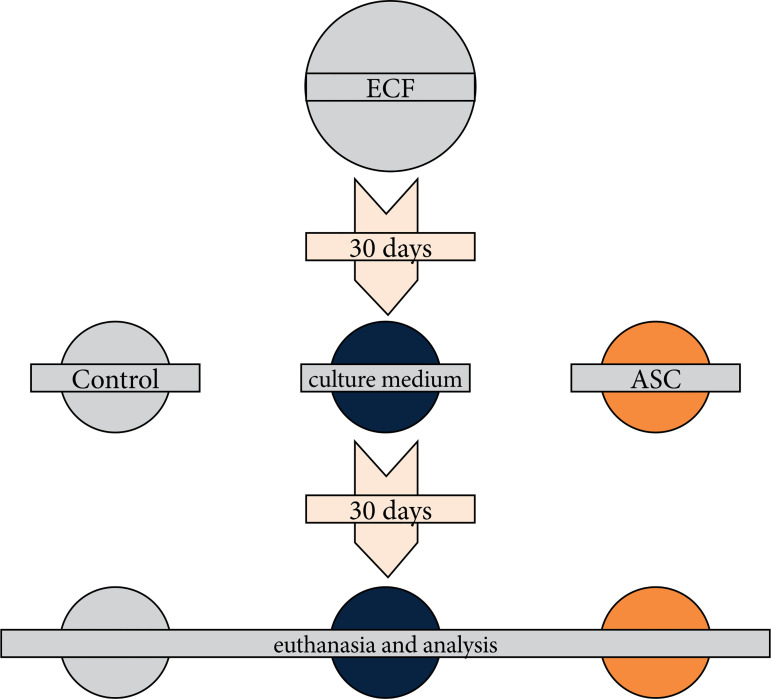
Experimental model flowchart: animals underwent the procedure to create an enterocutaneous fistula. After 30 days, they were randomly assigned to three groups: control, culture medium, and adipose tissue-derived stem cell. After another 30 days, the animals were analyzed and euthanized.

ECF: enterocutaneous fistula; ASC: adipose tissue-derived stem cell. Source: Elaborated by the authors.

After a 30-day recovery period, the animals were randomly divided into three groups: control (C), culture medium (CM), and ASC. The intervention was then performed, and after another 30 days, the effects of the treatment were evaluated (Fig. 2).

### Perifistular application of adipose tissue-derived stem cells

The animals in the culture medium and ASC groups were anesthetized using 2% isoflurane (Sigma-Aldrich, Germany). In the culture medium group, a perifistular injection of 0.5 mL of culture medium solution was administered. On the other hand, animals in the ASC group received a perifistular injection of 1 × 10^6^ ASC in 0.5 mL of solution.

### Macroscopic evaluation

To monitor the animals’ general condition, body weight and fistula output weekly examinations were conducted. The diameter of the ECF was measured using a caliper following the American Gastroenterological Association’s recommendation at the 30th postoperative day^30^.

### Histopathological analysis

ECF samples were collected and analyzed using optical microscopy (Nikon, United States of America) after staining with hematoxylin-eosin. The degree of fibrosis and inflammation was evaluated by analyzing 10 fields per slide at 40x magnification and graded on a 5-point scale, with 0 being normal intestinal epithelium, and 5 indicating high levels of fibrosis and abnormal infiltration of inflammatory cells.

### Statistical analysis

Continuous variables with normal distribution were expressed as mean and standard deviation, while continuous variables with non-normal distribution were presented as median and interquartile range. The analysis of variance (ANOVA) test was utilized for parametric variables, while the Kruskal-Wallis’ test was used for non-parametric variables in inferential analysis to compare groups. If significance was observed, a Bonferroni’s post hoc test was performed for pair-wise analysis. Statistical analysis was performed using the STATA version 14 program, with the significance level (alpha) of 5% and 80% study power.

## Results

All 21 animals that underwent the surgical procedure for ECF survived until the end of the analysis period.

### Macroscopic analysis

**Figure 3:**
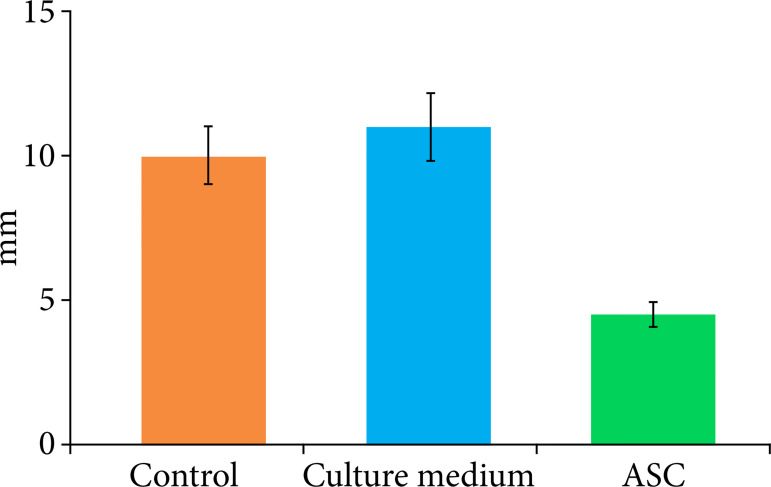
The diameter of the enterocutaneous fistula was compared among the control, culture medium, and adipose tissue-derived stem cell (ASC) groups. Bonferroni’s post hoc test revealed a significant reduction in the enterocutaneous fistula diameter in the ASC group compared to the control and culture medium groups (p < 0.05).

Source: Elaborated by the authors.

Animals that received ASCs (4.5 ± 1.4 mm) showed significant reduction of 55% in the diameter of ECF compared to the control group (10.0 ± 2.1 mm) and reduction of 59.1% compared to the culture medium group (11.0 ± 4.3 mm) (p = 0.003) (Fig. 3).

### Histopathological analysis

Table 1 presents the inflammation and fibrosis scores of the ECF tissue. The ASC group showed 21% less fibrosis compared to the control group, while there was no significant difference in the inflammatory score among the groups.

**Table 1:** Comparison of inflammation and fibrosis scores in the control, culture medium and adipose tissue-derived stem cell groups.

Group	Fibrosis score	Inflammation score
	Median (IQ)	Median (IQ)
Control	4.3 (3–5)	3.7 (3–4)
Culture medium	4.2 (4–5)	4.2 (4–5)
Adipose tissue-derived stem cells	3.4 (2–4)	3.2 (3–5)
P-value*	0.03	0.09

IQ: interquartile range;

* Bonferroni’s post hoc test. Source: Elaborated by the authors.

## Discussion

The study found significant reduction in the ECF diameter of the ASC group compared to both the control group (55% reduction, p = 0.001) and the culture medium group (55.9% reduction, p = 0.003). The ECF diameter was measured as 4.5 ± 1.4 mm in the ASC group, 10.0 ± 2.1 mm in the control group, and 11.0 ± 4.3 mm in the culture medium group. This indicates that ASC treatment has the potential to be effective in treating ECF diameter. It is worth noting that in this study all animals in the ASC group received a single injection session.

Regarding the histological analysis, the ASC group (3.4 IQ 2–4) demonstrated a 21% reduction in fibrosis compared to both the control group (4.3 IQ 3–5) and the culture medium group (4.2 IQ 4-5) (p = 0.03).

Adipose tissue is a promising source of stem cells due to its abundance, subcutaneous location, and less invasive harvesting technique^31^,^32^. We hypothesized that the use of ASC induces regenerative effects through paracrine effects. According to literature data, ASCs are known to produce a wide range of molecules that mediate inflammatory and immune response^22^. Thus, the reduction of inflammatory scenarios by ASCs can potentially benefit the wound healing process.

Consistent with current knowledge of mesenchymal cell therapies, their effects primarily result from paracrine activity rather than direct cellular differentiation. In another experimental study focusing on the effects of ASC on ECF, in which the cells were marked with bioluminescence, a 50% signal loss was observed after two days, as well as a 75% loss after 30 days, suggesting either cellular differentiation or loss of cell viability^33^. However, animals that exhibited a greater reduction in ECF also showed a stronger signal, indicating that multiple applications may be necessary to achieve the optimal cellular quantity.

One potential approach to enhance the survival of ASCs involves their concomitant application with an acellular matrix. The conventional cell injection method encounters challenges when applied to local regions due to significant cell loss and low cell survival rates. In contrast, cell sheet technology offers several advantages, as the adhesion proteins present in the sheet facilitate attachment to the tissue surface, and the cell-cell interactions within the sheet mimic physiological tissue behavior^34^. Notably, the co-administration of ASCs with a matrix has exhibited promising outcomes in experimental models of digestive system fistulas, such as perianal^35^ and pancreatic^34^,^36^ fistulas. Researchers are currently investigating various substances, including mannose, L-Lactide-Co-Caprolactone polymers, and hydrogels, as potential components of the ideal matrix. Nevertheless, successful development of these tissue bioengineering strategies necessitates ensuring the preservation of ASC viability and promoting seamless integration, while also minimizing potential damage to native tissues that could arise from contact with the matrix^37^.

In the context of wound healing physiology, the chronic state of ECF has been observed to involve a population of M1 macrophages, which can sustain the chronic inflammatory process and delay wound healing^38^. The alteration in the macrophage phenotypic profile may be one of the causes of immune response modulation. In an experimental study using a model of chronic wound in diabetes, the application of ASC increased the infiltration of M2-type macrophages, despite a reduction in the overall number of inflammatory cells present^39^.

In this study, we investigated the potential of ASC treatment in providing an anti-inflammatory stimulus for conditions such as inflammatory bowel diseases and other sources with high levels of tumor necrosis factor (TNF). TNF is a proinflammatory cytokine that induces the production of interleukin-1 (IL-1) and IL-6 cytokines, increases leukocyte mobility, and enhances capillary permeability, leading to a continuous inflammatory response that can hinder tissue healing. Therefore, therapies that can target this mechanism, such as administration of ASC, have the potential to facilitate the tissue healing process^2^,^40^,^41^.

The application of mesenchymal stem cells has already shown effects on modulating the inflammatory response. The upregulation of Sox6, Col2a1, and Agc1 expression in a murine model of bronchopleural fistula may be associated, respectively, with stem cell activation and differentiation, modulation of the inflammatory response, and increased proteolytic activity in tissue remodeling^42^. In a rat model study of radiation-induced proctitis, the injection of ASC demonstrated reduction in fibrosis and an increase in proliferation, aligning with our findings^43^. The Ki-67 stain revealed a higher presence of proliferative cells in the ASC injection groups, suggesting its potential to support epithelial regeneration. Moreover, the observed changes in caspase-3 positive cells in the mucosa indicated alterations in radiation-induced apoptosis mechanisms.

While research and technology on ASC have advanced, additional investigations are required to standardize protocols, define indications and administration systems, and confirm safety and effectiveness before considering ASC therapy for clinical use. To date, 18 preclinical studies and four review papers have demonstrated promising results in terms of safety and positive effects on morphology, function, and clinical outcomes, primarily in rat and porcine models^44^. However, the variability in study designs, models, interventions, and outcome measures has made it impractical to conduct a reliable meta-analysis.

This study has some limitations, including the use of a murine model and a small sample size.

## Conclusion

This study suggests that the use of ASC could be a promising therapeutic strategy for treating ECF. The treatment showed reduction in fistula diameter and tissue fibrosis.

## Data Availability

The data will be available upon request.
